# Detection of Oxacillin Resistance in *Staphylococcus aureus* Isolated from the Neonatal and Pediatric Units of a Brazilian Teaching Hospital

**DOI:** 10.4137/cmped.s2085

**Published:** 2009-03-18

**Authors:** Valéria Cataneli Pereira, André Martins, Lígia Maria Suppo de Souza Rugolo, Maria de Lourdes Ribeiro de Souza da Cunha

**Affiliations:** 1 Department of Microbiology and Immunology—Botucatu Biosciences Institute, UNESP, Brazil.; 2 Department of Pediatrics—Botucatu Medical School, UNESP, Brazil.

**Keywords:** *Staphylococcus aureus*, oxacillin, *mecA*, Pediatrics, NICU

## Abstract

**Objective:**

To determine, by phenotypic and genotypic methods, oxacillin susceptibility in *Staphylococcus aureus* strains isolated from pediatric and neonatal intensive care unit patients seen at the University Hospital of the Botucatu School of Medicine.

**Methods:**

A total of 100 *S. aureus* strains isolated from the following materials were studied: 25 blood cultures, 21 secretions, 12 catheters, 3 cannulae and one chest drain from 62 patients in the neonatal unit, and 36 blood cultures, one pleural fluid sample and one peritoneal fluid sample from 38 patients in the pediatric unit. Resistance of the *S. aureus* isolates to oxacillin was evaluated by the disk diffusion method with oxacillin (1 μg) and cefoxitin (30 μg), agar screening test using Mueller-Hinton agar supplemented with 6 μg/ml oxacillin and 4% NaCl, and detection of the *mecA* gene by PCR. In addition, the isolates were tested for β-lactamase production using disks impregnated with Nitrocefin and hyperproduction of β-lactamase using amoxicillin (20 μg) and clavulanic acid (10 μg) disks.

**Results:**

Among the 100 *S. aureus* strains included in the study, 18.0% were resistant to oxacillin, with 16.1% MRSA being detected in the neonatal unit and 21.0% in the pediatric unit. The oxacillin (1 μg) and cefoxitin (30 μg) disk diffusion methods presented 94.4% and 100% sensitivity, respectively, and 98.8% specificity. The screening test showed 100% sensitivity and 98.8% specificity. All isolates produced β-lactamase and one of these strains was considered to be a hyperproducer.

**Conclusions:**

The 30 μg cefoxitin disk diffusion method presented the best result when compared to the 1 μg oxacillin disk. The sensitivity of the agar screening test was similar to that of the cefoxitin disk diffusion method and higher than that of the oxacillin disk diffusion method. We observed variations in the percentage of oxacillin-resistant isolates during the study period, with a decline over the last years which might be related to improved nosocomial infection control and the rational use of antibiotics.

## Introduction

The genus *Staphylococcus* comprises about 40 species, with *Staphylococcus aureus* being the main representative and the causative agent of a wide variety of infections. Nosocomial infections are the main cause of morbidity and mortality in pediatric (PICU) and neonatal intensive care units (NICU).

Antimicrobial resistance is easily acquired by these microorganisms because of the high risk of plasmid transfer between strains in the hospital environment and the abusive use of antimicrobial drugs, which represent important factors for the transfer of resistance genes and the selection of multiresistant strains.^1^,^2^ The prevalence of oxacillin-resistant *S. aureus* (MRSA) in hospitals has increased in most countries. The intrinsic resistance of *S. aureus* to oxacillin is due to the production of a supplemental penicillin-binding protein (PBP 2’ or PBP 2a), which presents low affinity for semi-synthetic penicillins and is encoded by the chromosomal gene *mecA*. Although the resistance of *mecA* gene is present in all the cells of the population with intrinsic resistance, this could be expressed by a small percentage of them, leading to what has been called heterogeneous resistance. Other resistance mechanisms have been described in strains that do not possess the *mecA* gene, with these strains being called borderline resistant. Borderline resistance is caused by two mechanisms: inactivation of oxacillin due to β-lactamase hyperproduction^3^ and modified resistance, known as MOD-SA, due to the production of modified intrinsic PBPs with affinity for altered oxacillin.^4^

The reference methods for the detection of oxacillin resistance in *S. aureus* recommended by the Clinical and Laboratory Standards Institute (CLSI)^5^ include the determination of the minimum inhibitory concentration (MIC) of the drug by the agar or broth dilution method, disk diffusion method and, more recently, the cefoxitin disk diffusion test.^5^,^6^

Since phenotypic methods for the detection of MRSA may yield questionable results, molecular tests for the detection of the *mecA* gene or its product PBP 2a have been proposed. Detection of the *mecA* gene by the polymerase chain reaction (PCR) is considered to be the gold standard for the diagnosis of MRSA. The determination of oxacillin resistance in *Staphylococcus* is important to guide therapy and to prevent unnecessary treatment of the patient with vancomycin, an antibiotic associated with therapeutic complications and whose use may lead to the selection of resistant strains. The prevalence of MRSA presents varies widely, particularly as a function of the size and type of the health institution. Thus, the objective of the present study was to determine oxacillin susceptibility in *S. aureus* strains isolated from patients hospitalized in the PICU and NICU of the University Hospital of the Botucatu School of Medicine (HC-FMB) using the oxacillin and cefoxitin disk diffusion methods. These methods were compared to *mecA* gene detection by PCR.

## Materials and Methods

### Strains

A total of 100 *S. aureus* strain isolated from the following materials were studied: 25 blood cultures, 21 secretion, 12 catheters, 3 cannulae and one chest drain from 62 patients hospitalized in the NICU of HC-FMB, and 36 blood cultures, one pleural fluid sample and one peritoneal fluid sample from 38 patients hospitalized in the PICU between 1991 and 2007. The strains were isolated as described by Koneman et al.^7^

### Detection of oxacillin resistance by the 1 μg oxacillin and 30 μg cefoxitin disk diffusion method and by the agar screening test using Mueller-Hinton agar supplemented with 4% NaCl and 6 μg/mL oxacillin

Oxacillin sensitivity was tested by the agar disk diffusion method according to the criteria of the CLSI.^5^ The inocula were prepared in brain-heart infusion (BHI) broth and incubated for 4 to 6 h and turbidity was set to 0.5 McFarland standard. The following disks were used: 1 μg oxacillin and 30 μg cefoxitin. Once the density was adjusted, the inoculum was spread with a sterile swab on Mueller-Hinton agar and the disks impregnated with the drug were applied. The plates were incubated for 24 h at 35 °C and antimicrobial activity was evaluated by determining the diameter of the inhibition zone as recommended by the CLSI.^5^ The *S. aureus* ATCC 25923 (negative control) and ATCC 33591 (positive control) reference strains were used in all experiments.

For the detection of MRSA, Mueller-Hinton agar plates containing 6 μg/ml oxacillin and 4% NaCl were used.^8^ The inoculum was prepared by previous incubation in BHI broth for 24 h and turbidity was set to 0.5 McFarland standard. After preparation of the inoculum, the strains were seeded in spots on the agar surface with a sterile swab. The plates were incubated for 24 h at 35 °C and the presence of MRSA was defined as the growth of at least one colony on the agar surface.

### Detection of the *mecA* gene by PCR

Total nucleic acids were extracted from *S. aureus* strains cultured on blood agar, individually inoculated into BHI broth and incubated for 24 h at 37 °C. DNA was extracted using the GFX kit (Amersham Pharmacia Biotech). Briefly, staphylococcal cells were first digested with 10 μg/ml lysozyme and 20 μg/ml proteinase K. Next, 500 μl of the extraction solution was added and the mixture was centrifuged at 5000 × g for 1 min. The supernatant was then transferred to a GFX column and centrifuged at 5000 × g for 1 min. The collected fluid was discarded and 500 μl of the extraction solution was again added to the column. After centrifugation and discarding the collected fluid, 500 μl of the washing solution was added to the column which was centrifuged at 14,000 × rpm for 3 min. The column was then transferred to a 1.5-ml tube and 200 μl Milli-Q water heated to 70 °C was used for elution. The isolates were centrifuged at 5000 × g for 1 min and the GFX column was discarded. The extracted DNA was stored in a refrigerator at 4 °C.

PCR was carried out in 0.5-ml microcentrifuge tubes in a total volume of 25 μl containing 1 μM of each primer, 2.0 U Taq polymerase, 100 μM deoxyribonucleotide triphosphates, and 150 μg nucleic acid. The following primers were used: *mecA1* (forward)—5′ AAA ATC GAT GGT AAA GGT TGG 3′, and *mecA2* (reverse) – 5′ AGT TCT GCA GTA CCG GAT TTG 3′. The size of the amplified product is 533 bp. PCR was carried out in an appropriate thermocycler using the following parameters as described by Murakami et al.^9^ 40 cycles of denaturation at 94 °C for 30 s, annealing at 55.5 °C for 30 s and extension at 72 °C for 1 min. After the 40 cycles, the tubes were incubated at 72 °C for 5 min before cooling to 4 °C. International reference strains were used as positive (*S. aureus* ATCC 33591) and negative controls (*S. aureus* ATCC 25923) in all tests.

The efficiency of the amplification reactions was evaluated by electrophoresis on 2% agarose gel prepared in 0.5X TBE buffer and stained with ethidium bromide. The size of the amplified products was compared with the 100-bp standard and the gels were photographed under UV illumination.

### Determination of β-lactamase production and β-lactamase hyperproduction

The production of β-lactamase by the *S. aureus* isolates was tested using disks impregnated with Nitrocefin (chromogenic cephalosporin, cefinase, BBL). The disk was moistened with one or two drops of sterile distilled water and placed on the *S. aureus* colonies previously incubated for 24 h at 35 °C on the Mueller-Hinton plates used for the disk diffusion test with oxacillin (1 μg) and cefoxitin (30 μg), because these drugs stimulate β-lactamase production in *Staphylococcus*.

A positive reaction was indicated by the development of a red color, whereas the absence of a change in color indicated a negative reaction. For β-lactamase-negative strains, the reaction was reexamined after 1 h according to manufacturer recommendations. For correct analysis of the results, the tested disks were compared with positive (*S. aureus* ATCC 29213) and negative (*S. xylosus* ATCC 29979) control strains.

The isolate that was negative for the *mecA* gene and presented resistance to oxacillin by the phenotypic methods was tested to determine whether it was a hyperproducer of β-lactamase. This strain was tested using disks containing amoxicillin (20 μg) and clavulanic acid (10 μg). The sensitivity breakpoint was the formation of an inhibition zone ≥20 mm after incubation for 24 h at 35 °C.^10^

### Statistical analysis

Sensitivity and specificity tests^11^ were performed to compare the agar screening method, disk diffusion test with cefoxitin and oxacillin and PCR. The last method is considered to be the gold standard for the detection of intrinsic oxacillin resistance (detection of the *mecA* gene).

Sensitivity was defined as the proportion of PCR-positive *S. aureus* isolates (detection of the *mecA* gene) that were resistant to oxacillin using the following phenotypic methods: disk diffusion with cefoxitin and oxacillin, and agar screening test (Mueller-Hinton agar supplemented with 6 μg/mL oxacillin and 4% NaCl).

Specificity was defined as the proportion of PCR-negative *S. aureus* isolates (no detection of the *mecA* gene) that were sensitive to oxacillin using the following phenotypic methods: disk diffusion with cefoxitin and oxacillin, and agar screening test (Mueller-Hinton agar supplemented with 6 μg/mL oxacillin and 4% NaCl).

## Results

### Detection of oxacillin resistance by the 1 μg oxacillin and 30 μg cefoxitin disk diffusion method and by the agar screening test using Mueller-Hinton agar supplemented with 4% NaCl and 6 μg/mL oxacillin

**Figure 1:**
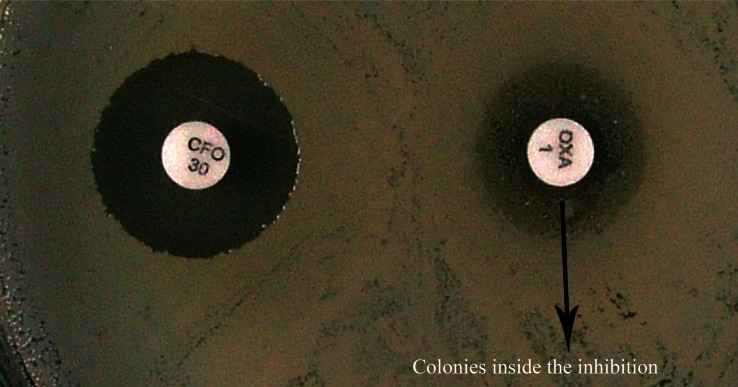
Strain resistant to cefoxitin and growth of colonies inside the inhibition zone, suggesting heterogenous resistance.

**Figure 2:**
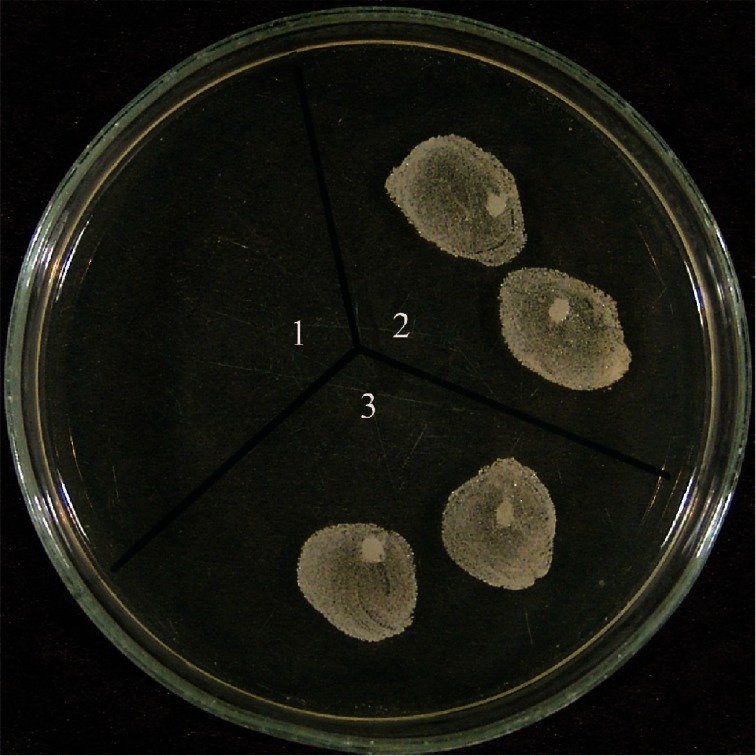
*Staphylococcus aureus* strains sensitive to oxacillin (1) and resistant to oxacillin (2 and 3) by the agar screening method (Mueller-Hinton agar containing 6 μg/mL oxacillin + 4% NaCl).

Among the 100 *S. aureus* strains isolated from PICU and NICU patients of HC-FMB, 11 (11%) were found to be resistant to oxacillin in the 1 μg oxacillin disk diffusion test. In seven (7%) isolates, growth of colonies was observed inside the inhibition zone, suggesting heterogenous resistance. Using 30 μg cefoxitin disks, oxacillin resistance was observed in 18 (18%) isolates and one (1%) presented growth of colonies inside the inhibition zone, suggesting heterogeneous resistance (Fig. 1). Nineteen (19%) isolates were found to be resistant by the agar screening method using *S. aureus* ATCC 33591 and ATCC 25923 as control strains (Fig. 2).

### Detection of the *mecA* gene by PCR

**Figure 3:**
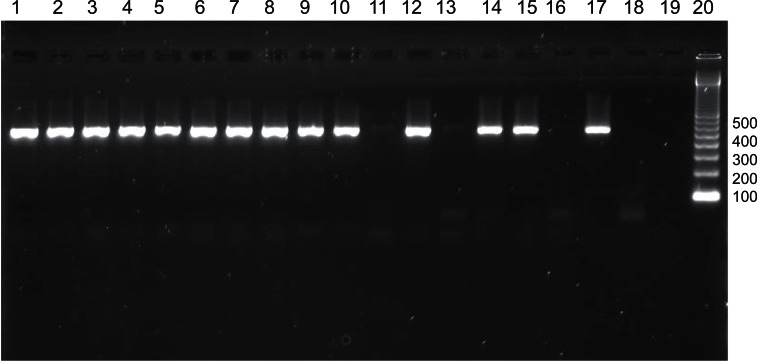
Agarose gel electrophoresis for the detection of the *mecA* gene (533 bp) in *Staphylococcus aureus* strains by PCR. Lanes 1, 2, 3, 4, 5, 6, 7, 8, 9, 10, 12, 14, and 15: positive strains; lanes 11, 13, and 16: negative strains; lane 17: positive control (*S. aureus* ATCC 33591); lane 18: negative control (*S. aureus* ATCC 25923); lane 19: water; lane 20: molecular weight marker (100 bp).

PCR revealed the presence of the *mecA* gene in 18 (18%) isolates. Eleven of these isolates were resistant in the 1 μg oxacillin and 30 μg cefoxitin disk diffusion tests and one isolate only showed resistance in the 30 μg cefoxitin disk diffusion test. Among the other six PCR-positive isolates, five presented heterogenous resistance by the oxacillin disk diffusion method and resistance by the cefoxitin disk diffusion method. One isolate showed heterogenous resistance with both disk diffusion methods. All 18 *mecA*-positive isolates were found to be resistant in the agar screening test (Fig. 3 and Table 1).

### Determination of β-lactamase production and β-lactamase hyperproduction

The production of β-lactamase was determined using disks impregnated with Nitrocefin. All *S. aureus* isolates were confirmed to be producers of β-lactamase.

The isolate that was negative for the *mecA* gene, was resistant by the 30 μg cefoxitin disk diffusion and screening methods, and showed heterogenous resistance in the 1 μg oxacillin disk diffusion test was studied to determine whether it was a hyperproducer of β-lactamase. This was tested with a disk of amoxicillin (20 μg) and clavulanic acid (1 μg) and was sensitive to this drug, then confirming the hyperproduction of β-lactamase, that was inhibited by the presence of clavulanic acid.

### Statistical analysis

Statistical analysis showed 94.4% sensitivity and 98.8% specificity of the oxacillin disk diffusion method and 100% sensitivity and 98.8% specificity of the cefoxitin disk diffusion method. The sensitivity and specificity of the screening test was 100% and 98.8%, respectively (Table 2).

### Evolution of oxacillin resistance in *S. aureus* strains isolated from patients seen at HC-FMB

Sixty-two samples from NICU patients and 38 from PICU patients were studied. Among the strains isolated from the neonatal unit, 10 (16.1%) were resistant to oxacillin, three of them isolated from blood cultures, 4 from secretions and 3 from catheters. Eight (21%) of the strains isolated from the PICU were MRSA, with 7 strains being isolated from blood cultures and one strain isolated from pleural fluid (Table 3).

**Figure 4:**
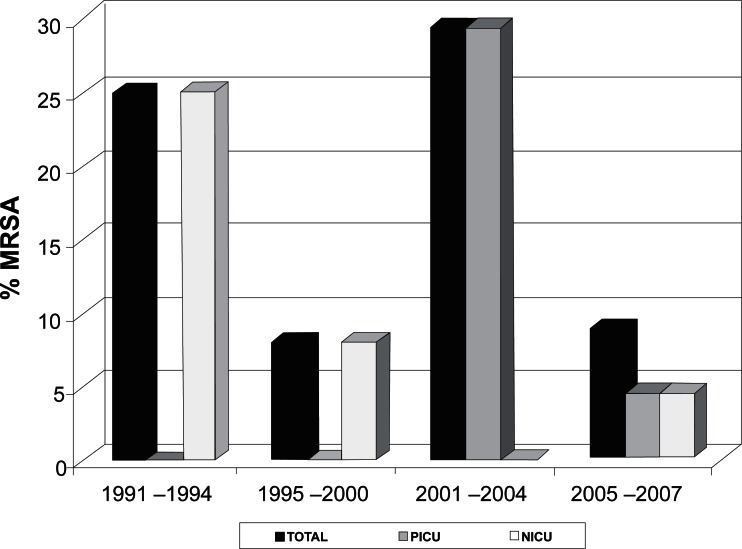
Evolution of oxacillin resistance in *Staphylococcus aureus* isolated at the University Hospital of the Botucatu School of Medicine between 1991 and 2007. PICU: pediatric intensive care unit; NICU: neonatal intensive care unit.

The strains of the present study were isolated between 1991 and 2007. Twenty-eight strains were isolated between 1991 and 1994, and seven (25%) were found to be resistant to oxacillin. Twenty-five strains were isolated between 1995 and 2000, and two (8%) were MRSA. Of the 24 strains isolated between 2001 and 2004, seven (29.2%) were MRSA. Twenty-three strains were isolated between 2005 and 2007, and two (8.7%) were resistant to oxacillin (Fig. 4).

The seven (25%) MRSA strains isolated between 1991 and 1994 and the two (8%) MRSA strains isolated between 1995 and 2000 originated from the neonatal unit. In contrast, the seven (29.2%) MRSA strains isolated between 2001 and 2004 were from children of the pediatric unit. Of the two oxacillin-resistant strains isolated between 2004 and 2007, one (4.4%) was from the neonatal unit and the other (4.4%) was from the pediatric unit (Table 4 and Fig. 4).

## Discussion

The prevalence of oxacillin-resistant *S. aureus* in hospitals has increased in most countries. Prevalence rates of MRSA may vary, particularly as a function of the size and type of the medical institution. In the present study, 100 *S. aureus* strains isolated from inpatients of the PICU and NICU of HC-FMB were tested for MRSA.

Although high frequencies of oxacillin-resistant *S. aureus* have been reported especially in large hospitals and university hospitals, in the present study the percentage of MRSA isolated from inpatients of the PICU and NICU of HC-FMB between 1991 and 2007 was only 18%. Similar results have been reported for PICU and NICU patients with bacteremia in the United Kingdom, with a percentage of MRSA of 15.5%.^12^ Furthermore, the prevalence of MRSA observed here was lower than that reported in studies conducted in other countries. In the United States, a prevalence of MRSA infection of 47% was reported in a hospital in Texas in 2003.^13^ A study conducted in India found a prevalence of oxacillin-resistant *S. aureus* of 66% among NICU inpatients.^14^ Similar rates have been reported in studies conducted in Japan and Israel, with MRSA infection rates of 52.5% and 60%, respectively, among inpatients of an NICU.^15^,^16^

In the present study, phenotypic tests such as the disk diffusion method and agar screening test were compared with a genotypic method (*mecA* gene detection by PCR). The cefoxitin disk diffusion method presented 100% sensitivity and 98.8% specificity and was superior to the oxacillin disk diffusion method (94.4% sensitivity and 98.8% specificity) in terms of the detection of oxacillin-resistant *S. aureus*. Similar results have been reported by Velasco et al.^17^ According to these authors, the 30 μg cefoxitin disk diffusion method, recently recommended by the NCCLS^6^ and CLSI^5^ as a screening test, was the best method showing 100% sensitivity and 98% specificity. Recent studies evaluating cefoxitin disks for the detection of MRSA also obtained good results, with a sensitivity of about 100% and specificity of 99%.^18^–^21^ Cauwelier et al.^20^ evaluated methicillin resistance in 155 clinical MRSA isolates by different methods including oxacillin and cefoxitin disks, latex agglutination and an agar screening test. The cefoxitin disk diffusion method presented 100% sensitivity and 99% specificity, whereas sensitivity fell to 91.7% in the oxacillin disk diffusion test. According to these authors, compared to the gold standard (*mecA* gene detection), the disk diffusion method with 30 μg cefoxitin is preferable to the 1 μg oxacillin disk diffusion method for the detection of MRSA.

The *mecA* gene was detected in all isolates that were resistant in the Mueller-Hinton agar screening test. The sensitivity of the method was 100% and specificity was 98.8%. The performance of the screening method using Mueller-Hinton agar supplemented with 4% NaCl and 6 μg oxacillin depends on the degree of heterogeneity of the isolates tested, with lower sensitivity (≤95%) being reported in studies including a larger number of heteroresistant strains and values >97% in other studies.^22^–^24^ In this investigation, the sensitivity of the agar screening test for the detection of MRSA was similar to that of cefoxitin disks and higher than that of the oxacillin disk diffusion method. The high sensitivity of the agar screening method might be explained by the small number of heteroresistant strains. Different results have been reported by Cauwelier et al.^20^ who observed 91.7% sensitivity and 100% specificity. The authors attributed the low sensitivity in the detection of heterogeneous populations to the high variability in the expression of PBP 2a.

Other resistance mechanisms have been described in strains that do not possess the *mecA* gene, with these strains being called borderline resistant. There are two mechanisms of borderline resistance, one is the inactivation of oxacillin due to hyperproduction of β-lactamase^3^ and the other is modified resistance, called MOD-SA, due to the production of modified intrinsic PBPs with affinity for altered oxacillin.^4^ However, these mechanisms are characterized by low levels of resistance (MIC of 8 μg/ml).^24^ In the present study, one isolate presented resistance to oxacillin in all phenotypic tests despite the absence of the *mecA* gene and was sensitive to the amoxicillin-clavulanic acid disk. This strain was therefore considered to be a hyper-producer of β-lactamase, suggesting borderline resistance.

The MRSA strains detected during the first two study periods (1991 to 2000) were isolated from samples originating from the neonatal unit. This finding can be explained by the fact that all strains studied until 1998 exclusively originated from the neonatal unit and strains from the pediatric unit were only included after 1999. Between 2001 and 2004, all oxacillin-resistant strains originated from the pediatric unit, with a decline in the number of MRSA originating from the NICU. In contrast, in the last period the proportion of MRSA originating from the NICU and PICU was the same. Overall analysis of our data showed variations in the percentage of oxacillin-resistant *S. aureus* during the study period. The percentage of resistant isolates declined between 1995 and 2000, followed by an increase from 2001 to 2004, and then declined again over the following years. These reductions might be related to improved nosocomial infection control and the rational use of antibiotics.

## Figures and Tables

**Table 1:** Presence or absence of the *mecA* gene in strains tested by the oxacillin and cefoxitin disk diffusion method and the agar screening method.

**PCR^a^**	**Phenotypic test**					

	**Disk diffusion**				**Screening method^b^**	

	**Oxacillin (1 μg)**		**Cefoxitin (30 μg)**			

	**S**	**R**	**S**	**R**	**S**	**R**
*mecA* + (N = 18)	1	17	0	18	0	18
*mecA* − (N = 82)	81	1	81	1	81	1
**Total** (N = 100)	82	18	81	19	81	19

a Polymerase chain reaction.

b Mueller-Hinton agar containing 6 μg/mL oxacillin + 4% NaCl.

S Oxacillin-sensitive sample.

R Oxacillin-resistant sample.

**Table 2:** Determination of the sensitivity and specificity of phenotypic and genotypic methods for the detection of oxacillin resistance in *S. aureus* strains.

**Phenotypic test**	***MecA***		**Sensitivity**	**Specificity**
	
	**Positive (N = 18)**	**Negative (N = 82)**	**%**	**%**
Oxacillin disk (1 μg)	17	81	94.4	98.8
Cefoxitin disk (30 μg)	18	81	100	98.8
Screening method^a^	18	81	100	98.8

a Mueller-Hinton agar containing 6 μg/mL oxacillin + 4% NaCl.

**Table 3:** Determination of oxacillin resistance in *Staphylococcus aureus* strains according to hospital unit and clinical material.

	**Neonatal unit**			**Pediatric unit**		
	**N NICU**	**N MRSA**	**%**	**N PICU**	**N MRSA**	**%**
**Blood culture** (N = 61)	25	3	12	36	7	19.5
**Secretion** (N = 21)	21	4	19	0	0	0
**Peritoneal fluid** (N = 1)	0	0	0	1	0	0
**Pleural fluid** (N = 1)	0	0	0	1	1	100
**Catheter** (N = 12)	12	3	25	0	0	0
**Cannula** (N = 3)	3	0	0	0	0	0
**Chest drain** (N = 1)	1	0	0	0	0	0

N, total number of strains.

N MRSA, number of oxacillin-resistant *S. aureus* strains.

N NICU, number of strains originating from the neonatal unit.

N PICU, number of strains originating from the pediatric unit.

**Table 4:** Number of oxacillin-resistant and sensitive *Staphylococcus aureus* strains according to the period studied.

	**MRSA**		**MSSA**		**Total**
	**NICU**	**PICU**	**NICU**	**PICU**	
1991–1994	7	0	21	0	28
1995–2000	2	0	20	3	25
2001–2004	0	7	4	13	24
2005–2007	1	1	7	14	23
Total	10	8	52	30	**100**

MRSA, oxacillin-resistant *S. aureus*.

MSSA, oxacillin-sensitive *S. aureus*.

NICU, neonatal intensive care unit.

PICU, pediatric intensive care unit.
